# The influence of predator community composition on photoprotective traits of copepods

**DOI:** 10.1002/ece3.8862

**Published:** 2022-04-24

**Authors:** Rebecca Oester, Ryan Greenway, Marvin Moosmann, Ruben Sommaruga, Barbara Tartarotti, Jakob Brodersen, Blake Matthews

**Affiliations:** ^1^ ETH Zürich, D‐USYS Zürich Switzerland; ^2^ Department of Fish Ecology and Evolution Eawag Swiss Federal Institute of Aquatic Science and Technology, Centre for Ecology, Evolution and Biogeochemistry Kastanienbaum Switzerland; ^3^ Department of Evolutionary Biology and Environmental Studies University of Zurich Zurich Switzerland; ^4^ Institute of Applied Microbiology University of Applied Sciences and Arts of Southern Switzerland Mendrisio Switzerland; ^5^ Division of Aquatic Ecology and Evolution Institute of Ecology and Evolution University of Bern Bern Switzerland; ^6^ Department of Ecology Lake and Glacier Ecology Research Group University of Innsbruck Innsbruck Austria

**Keywords:** Arctic charr, astaxanthin, carotenoid, *Leptodiaptomus minutus*, mycosporine‐like amino acids, Photoprotection, threespine stickleback, UVR

## Abstract

Trait expression of natural populations often jointly depends on prevailing abiotic environmental conditions and predation risk. Copepods, for example, can vary their expression of compounds that confer protection against ultraviolet radiation (UVR), such as astaxanthin and mycosporine‐like amino acids (MAAs), in relation to predation risk. Despite ample evidence that copepods accumulate less astaxanthin in the presence of predators, little is known about how the community composition of planktivorous fish can affect the overall expression of photoprotective compounds. Here, we investigate how the (co‐)occurrence of Arctic charr (*Salvelinus alpinus*) and threespine stickleback (*Gasterosteus aculeatus*) affects the photoprotective phenotype of the copepod *Leptodiaptomus minutus* in lake ecosystems in southern Greenland. We found that average astaxanthin and MAA contents were lowest in lakes with stickleback, but we found no evidence that these photoprotective compounds were affected by the presence of charr. Furthermore, variance in astaxanthin among individual copepods was greatest in the presence of stickleback and the astaxanthin content of copepods was negatively correlated with increasing stickleback density. Overall, we show that the presence and density of stickleback jointly affect the content of photoprotective compounds by copepods, illustrating how the community composition of predators in an ecosystem can determine the expression of prey traits that are also influenced by abiotic stressors.

## Graphical abstract

Photoprotective trait expression of copepods is mediated by the predator community composition. In the absence of fish, the concentration of photoprotective compounds is highest and reduced depending on the fish identity and density.

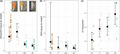

## 1 INTRODUCTION

Natural populations of prey species often experience stressful environmental conditions that are shaped by both multiple predators and abiotic conditions (Schmitz et al., 2017). These environmental stressors are often important ecological and evolutionary drivers of phenotypic variation, and can vary in their relative importance among populations across the landscape (Nussey et al., 2007). Understanding the mosaics of environmental stress (Gaynor et al., 2019) can help explain patterns of anti‐predator trait expression by prey (e.g., cryptic coloration and defensive morphology), and variation in the success of anti‐predator strategies, such as avoiding detection, surviving attacks, and growing beyond a vulnerable size (reviewed by Schmitz & Trussell, 2016). Anti‐predator traits and strategies of prey are often plastic and can be induced rapidly in response to predation risk (Dingemanse & Wolf, 2013), however, their expression is often conditional on prevailing abiotic conditions (Hansson, 2004).

A striking example of how natural populations respond to multiple environmental stressors is the regulation of photoprotective compounds (PCs) in freshwater copepods (Hairston, 1979; Hansson, 2000). Copepods can accumulate both carotenoids (e.g., astaxanthin) and mycosporine‐like amino acids (MAAs) from their algal diets, and thereby reduce their risk from ultraviolet radiation (UVR) (Rautio & Tartarotti, 2010). The accumulation of carotenoids causes most freshwater copepods to become bright red, and thus, more conspicuous to visual predators (Byron, 1982). In contrast, the accumulation of MAAs does not cause any red coloration. Copepods can likely regulate these alternative pathways depending on food availability (i.e., algal community), the opportunities for spatial refuge (e.g., vertical migration), and other abiotic and biotic conditions. Previous work has shown that the contents of MAAs and carotenoids of copepods can vary with respect to both UVR and predation risk (Brüsin et al., 2016; Garcia et al., 2014; Hylander, Larsson, et al., 2009). However, no previous study has examined how natural variation in the planktivorous fish community composition can affect carotenoid and MAA accumulation in copepods.

**FIGURE 1:**
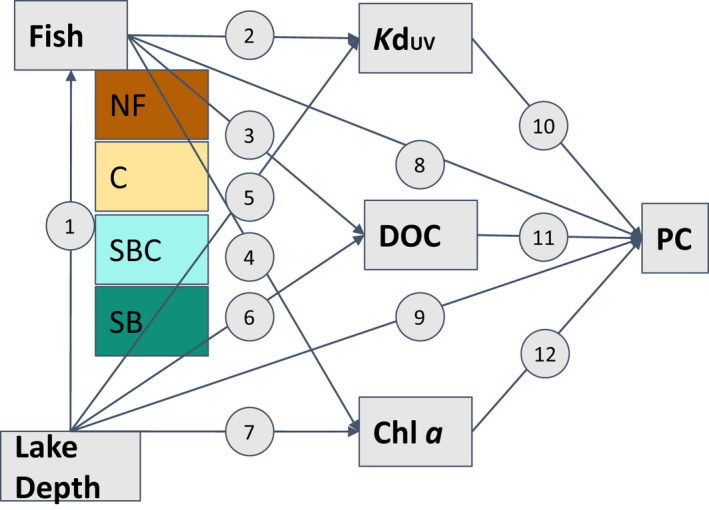
Base model with all possible direct and indirect paths between environmental factors and the photoprotective compounds (PCs). The fish variable is categorized in the four fish communities: NF, no fish; C, charr only; SBC, stickleback and charr; SB, stickleback only. The numbers refer to the hypotheses listed in Table 1

In the present study, we investigate how different predator communities consisting of threespine stickleback (*Gasterosteus aculeatus*) and/or Arctic charr (*Salvelinus alpinus*) affect the PCs of the copepod species *Leptodiaptomus minutus* in lakes in southern Greenland. Specifically, we asked the following questions: How does variation in lake environmental conditions help explain variation in PCs of *L*. *minutus* among lakes? How does the predator community of planktivorous fish species, i.e., stickleback and charr, affect PCs in *L*. *minutus*? To answer these questions, we sampled copepods from 73 lakes in Southern Greenland with different fish community structure and a wide variation in abiotic environmental conditions. We quantified both the mean contents of PCs among lakes (5–10 copepods aggregated), as well as intrapopulation variation in the astaxanthin contents by measuring PCs of individual copepods. Building on previous work on the putative causes of copepod coloration (Table 1), we developed a path model with a structured set of hypotheses about how environmental conditions and planktivore community composition can jointly affect copepods PCs (Figure 1). These hypotheses guided both our original data collection, as well as our subsequent analysis using Bayesian path analyses and generalized (non‐)linear multivariate multilevel models. Because there are only two freshwater fish species in southern Greenland lakes, our study is uniquely well suited to elucidate the species‐specific effects of predation risk on copepod accumulation of photoprotective compounds.

**TABLE 1:** Hypotheses that motivated the structure of the base model of the path analysis

Number	Hypothesis	Source
1	Lake depth can influence the fish community	Mehner et al. (2005), Wasserman et al. (2020)
2	Fish community can influence UV extinction (e.g., through bioturbation of sediments, or trophic cascades)	Mazumder et al. (1990), Adámek and Maršálek (2013)
3	Fish community can influence the concentration of DOC	Stief and Hölker (2006), Limberger et al. (2019)
4	Fish community can influence the concentration of chlorophyll *a*	Cañedo‐Argüelles et al. (2017)
5	Lake depth can influence UV extinction (e.g., through lake mixing)	Neale et al. (1998), Pérez‐Fuentetaja et al. (1999)
6	Lake depth can influence the concentration of DOC	Pérez‐Fuentetaja et al. (1999), Xenopoulos et al. (2003)
7	Lake depth can influence the concentration of chlorophyll *a*	Wagner et al. (2011)
8	Fish community can influence the accumulation of photoprotective compounds	This study
9	Lake depth can influence the accumulation of photoprotective compounds	Byron (1982), Tartarotti et al. (2004)
10	UV extinction can influence the accumulation of photoprotective compounds	Tartarotti et al. (2001), Tartarotti et al. (2017)
11	The concentration of DOC can influence the accumulation of photoprotective compounds (e.g., through water clarity)	Rautio and Tartarotti (2010)
12	The concentration of chlorophyll *a* can influence the accumulation of photoprotective compounds	Andersson et al. (2003)

## 2 MATERIAL AND METHODS

### 2.1 Study site

**FIGURE 2:**
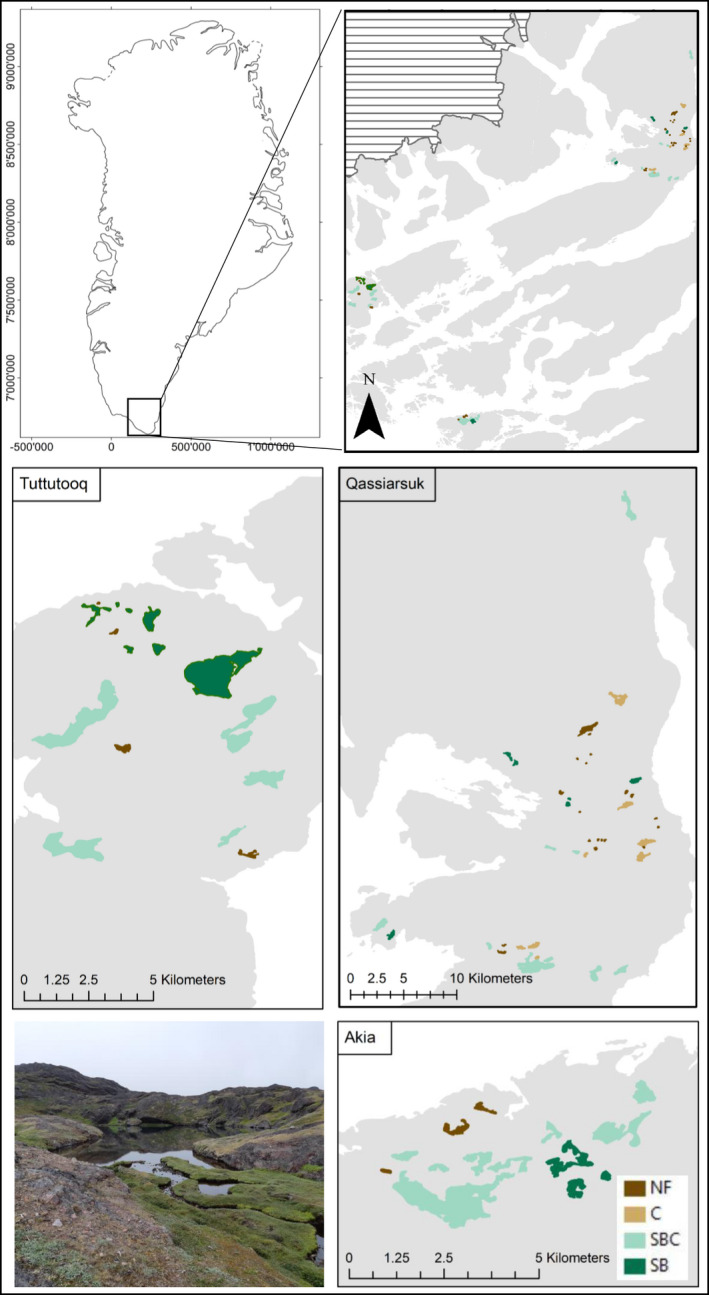
Maps and image of study sites in southern Greenland. NF, no fish; C, charr only; SBC, stickleback and charr; SB, stickleback only. The striped area represents ice cover. Base maps provided by GEUS in the coordinate system WGS 1984 World Mercator

We conducted a field survey in 73 lakes in southern Greenland (61°N, 46°W) around the Tunulliarfik Fjord (Qassiarsuk) and on nearby islands (Akia and Tuttuttoq) over the summer periods of 2018 and 2019 (Figure 2). In these regions, there are only two species of freshwater fish: threespine stickleback (*G*. *aculeatus*) and Arctic charr (*S*. *alpinus*). Hence, lakes could be grouped as follows: no fish (NF), charr only (C), stickleback and charr (SBC), and stickleback only (SB). The lakes were oligotrophic and clear (<5 µg/L chlorophyll *a*; 1.9–6.8 mg/L dissolved organic carbon (DOC); Table S1), and, in most lakes, the zooplankton community was dominated in abundance and biomass by the calanoid copepod species *L*. *minutus* (Table S2).

### 2.2 Field survey

For each lake in Greenland, we measured the physical and chemical properties to quantify the abiotic conditions relevant for the expression of PCs by copepods. We took profiles of photosynthetically active radiation (PAR; Li‐Cor LI‐1500) and UVR (~300–400 nm) from the lake surface to a depth of 2.5 m. Diffuse attenuation coefficients (*K*d) of UVR and PAR were calculated from the slope of the linear regression of the ln of the irradiance and depth. We took water samples from the upper 3 m using an integrated tube sampler (Tygon tubing; tube diameter 3 cm). The sampled water was filtered through GF/F filters (ashed for DOC at 450°C for 4 h; Whatman). For chlorophyll *a*, the filter was extracted in 1.1 ml ethanol (95%) for 24 h and measured with a spectrophotometer at 665 nm (Spectroquant NOVA 60A, Merck; ISO 10260, 1992). For DOC analysis, we acidified filtered lake water and 20 ml from each lake was analyzed with a total organic carbon analyzer (TOC‐L, Shimadzu). DOC samples from eight lakes in Akia were not included in the study due to contamination.

We sampled zooplankton communities from all 73 lakes with vertical net tows (net diameter: 25 cm; mesh size: 150 μm). Copepods were narcotized with CO_2_, picked with forceps (5–10 individuals per replicate: Copepodite V‐adult, excluding egg‐bearing females), and then transferred to HPLC autosampler vials (2.0 ml, cylindrical) with 1.0 ml 100% ethanol. In 24 lakes, we additionally transferred 20 individual copepods into separate HPLC autosampler vials (1.0 ml, 12 × 32 mm conical) with 0.75 μl ethanol. We standardized the contents of PCs by the dry weight of the copepod sample, estimated from the average prosome length of each population and a length–weight relationship (Lawrence et al., 1987). To estimate stickleback density, we set unbaited minnow traps along the shoreline of the lakes for on average of 3.5 h to achieve a minimum catch of 50 individuals. We calculated biomass per unit effort (BPUE: expressed as g trap^−1^ h^−1^) using length–biomass regression for stickleback (Pennycuick, 1971).

### 2.3 Photoprotective compounds

We analyzed carotenoids and MAAs of copepods with high‐performance liquid chromatography (HPLC). For the carotenoids to be fully extracted, the samples were stored in ethanol (100%) for at least 24 h. Fifty microliter of each sample extraction was injected in a LC‐4000 HPLC system containing a 5 μm pore size C18 column (LiChroCART 250‐4, Merck). The temperature in the column oven was set to 30°C and the flow rate of the mobile phase (45% ethyl acetate, 35% methanol, and 20% H_2_O) was set to 1.0 ml/min, with an entire chromatogram time of 10 min. Spectral absorbance was measured with a photodiode array detector (MD‐2018 Plus, Jasco). The different types of carotenoids were identified by comparing the relative retention times with other published reports, their spectra, and through chromatographic analyses with reference to standards (DHI).

For the analysis of MAAs, the samples were dried using a SpeedVac (SC110, Savant) and resuspended in 25% methanol before being sonicated on ice. The remaining steps were done as described in Tartarotti and Sommaruga (2002), with some modifications (Tartarotti et al., 2017). The detection limit in the HPLC for carotenoid peaks was an area of 1700 µV sec resulting in a content of 0.246 ng/µg dry weight. Half of this value was used for samples falling under the detection limit (44 out of 801 samples: 5%). For the HPLC for MAAs, the minimal observed MAA was detected at 0.05 ng/µg dry weight. Hence, the samples where no peaks could be detected received the level of 0.025 ng/µg dry weight (6 of 55 samples; 11%). For the MAA dataset, three outliers were excluded based on an analysis of Cook’s distance.

### 2.4 Statistical analyses

We conducted all calculations and statistical analyses in R‐studio (version 4.1.2; R Development Core Team, 2020). In a first step, we assessed the relative importance of relevant environmental factors to the three response variables (i.e., astaxanthin contents, MAA contents, and CV of astaxanthin) using Bayesian path analyses. Factors relevant to the light regime and prey refuge include lake depth, the attenuation coefficient of UV (*K*d_UV_), DOC, and chlorophyll *a*, whereas the predatory environment is represented by the presence/absence of the two planktivorous fish species (i.e., charr and stickleback). We began our analysis with a path model that included all possible direct and indirect paths, as shown in Figure 1. We scaled all numerical variables to have a mean around 0 and coded the fish community as four categories: No fish (reference), charr only, stickleback and charr, and stickleback only. We used non‐informative priors and either Gaussian (identity link) or categorical (logit link) families with the default settings provided by the brms package (Bürkner et al., 2021). We generated 20,000 (four chains run for 10,000 iterations with the first 5000 discarded as burn‐in) Markov chain Monte Carlo (MCMC) samples from the posterior distribution. Draws were sampled using NUTS (No‐U‐Turn Sampler). The MCMC chains showed convergence within the threshold specified by Gelman and Rubin (1992), meaning that the rhat statistic for all model parameters was close to 1. All models showed high effective sample size measures, also indicating convergence.

After identifying the main drivers of the variation in the PCs, we then assessed the effect of predator species presence and lake depth (including the interaction) on PCs among and within lakes using Bayesian regression analyses. We used Bayesian generalized (non‐)linear multivariate multilevel models with non‐informative priors for all further analyses. For the response variables astaxanthin and MAAs, we defined the family as a Gamma distribution with a log link function. As the CV of astaxanthin is a ratio and expected to lie within 0–1, we defined the priors as a uniform distribution (0,1) from a Gaussian family. We generated 4000 (four chains run for 2000 iterations with the first 1000 discarded as burn‐in) Markov chain Monte Carlo (MCMC) samples from the posterior distribution. We visually checked the fit of the posterior distribution with the data using the pp_check function from the bayesplot package (Gabry et al., 2021). We predicted the effects of predators and lake depth, and their interaction for each of the three response variables separately.

Then, we tested the individual effect of stickleback, expressed as BPUE, on astaxanthin, MAA, and CV of astaxanthin. Lastly, we investigated the relationship between the two PCs astaxanthin and MAAs. We specified the family of distributions and priors of these models in the same way as previously described. The posterior means and 95% credible intervals for relevant model parameters are presented and conditional effects plots were used to visualize the relationships between the response and predictor variables using the conditional_effects function in the brms package (Bürkner et al., 2021).

## 3 RESULTS

The PCs that we observed in *L*. *minutus* are the carotenoid astaxanthin, and six different MAAs: mycosporine‐glycine, shinorine, porphyra, asterina‐330, palythine, and an unknown MAA with an absorption peak at 332 nm. Palythine and shinorine were the most abundant MAAs and showed the highest contents (up to 10 ng/µg dry weight). We used the sum of all MAAs as our response variable in subsequent analyses, as the different MAAs fulfill the same functions at different wavelengths, and the total of MAAs is a useful measure of the overall photoprotection through this pathway (Shick & Dunlap, 2002).

**FIGURE 3:**
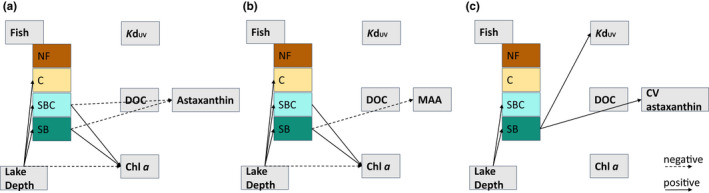
Results of the path model are shown for (a) astaxanthin, (b) MAA, and (c) CV of astaxanthin. Arrows are displayed when 0 was not included in the credible interval. Solid arrows represent positive estimates and dashed arrows show negative estimates. The exact values of the estimates and confidence intervals for each path can be found in Table S3. For abbreviations see Figure 1

The path analyses provided evidence that all three response variables (i.e., astaxanthin, MAA, and CV of astaxanthin) were associated with the fish community composition (Figure 3; Table S3). We found that low contents of astaxanthin were directly associated with fish communities that included stickleback (regardless of charr presence). Our data also suggest that there was an indirect effect of lake depth on fish community compositions, as the fishless lakes were on average shallower (Table S1). For MAAs, we found a direct negative association between fish communities that only included stickleback and an indirect effect of lake depth. The CV of astaxanthin was positively associated with the fish communities that only included stickleback. Other environmental factors such as *K*d_UV_, DOC, or chlorophyll *a* had no direct effect on the PCs.

**FIGURE 4:**
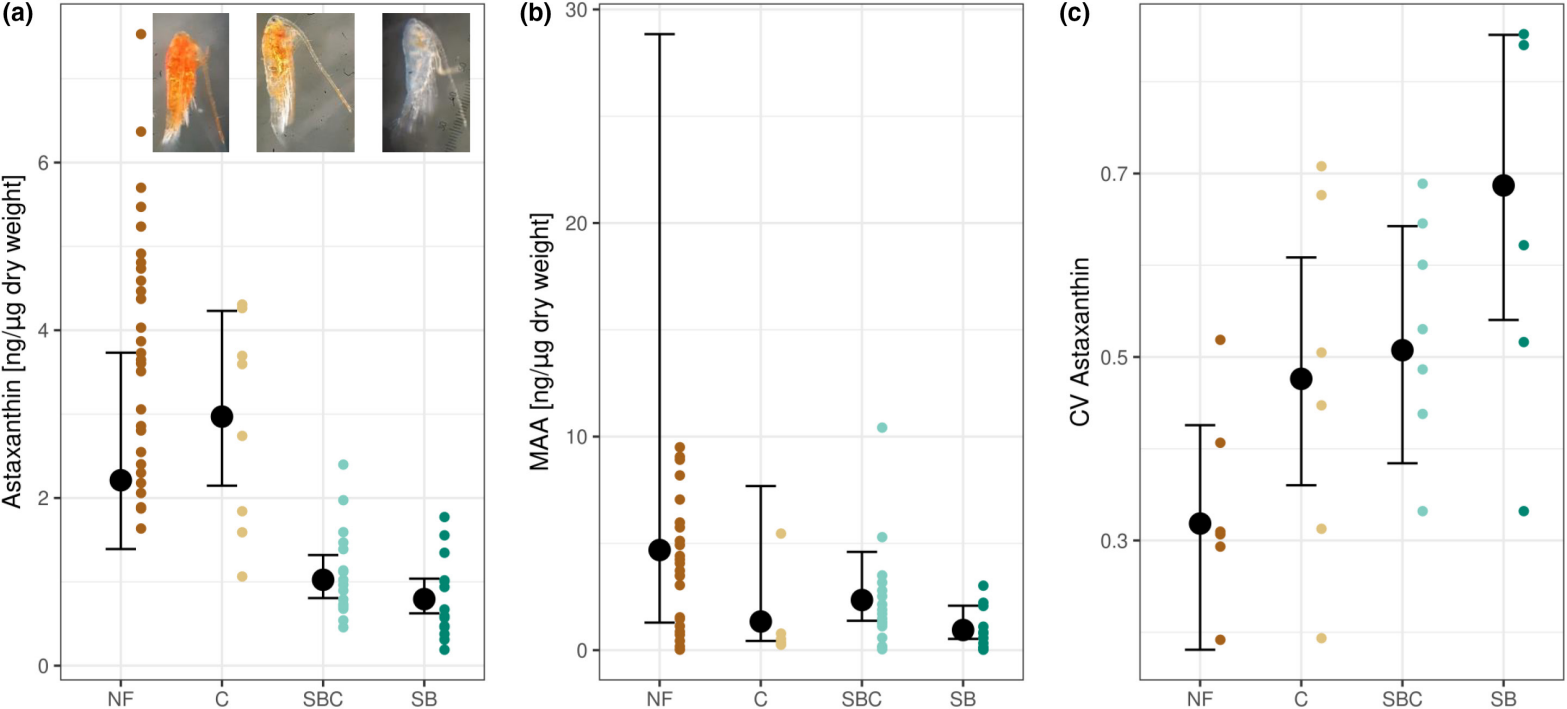
Effects of fish on (a) astaxanthin, (b) MAA, and (c) CV of astaxanthin. The colored dots represent the observed data points. The black dots represent posterior medians, and the error bars show 95% credible intervals for conditional effects at their mean lake depth. For abbreviations see Figure 1

We found evidence that the three response variables were mostly associated with the fish community (Figure 4; Table S4). Compared to fishless lakes, the astaxanthin contents were lower in lakes with stickleback (SBC: estimate: −1.74, CI [−2.24; −1.26]; SB: estimate: −1.77, CI [−2.31; −1.22]), and only in lakes including both fish species, the lake depth had a positive interactive effect (SBC: estimate: −0.15, CI [0.03; 0.28]). For MAAs, the credible intervals showed greater overlap, however, in lakes containing only stickleback, the copepods had the lowest MAAs contents (SB: estimate: −1.42, CI [−2.77; −0.03]). Stickleback also showed the largest effect in the CV of astaxanthin (SB: estimate: 0.18, CI [0.02; 0.42]).

**FIGURE 5:**
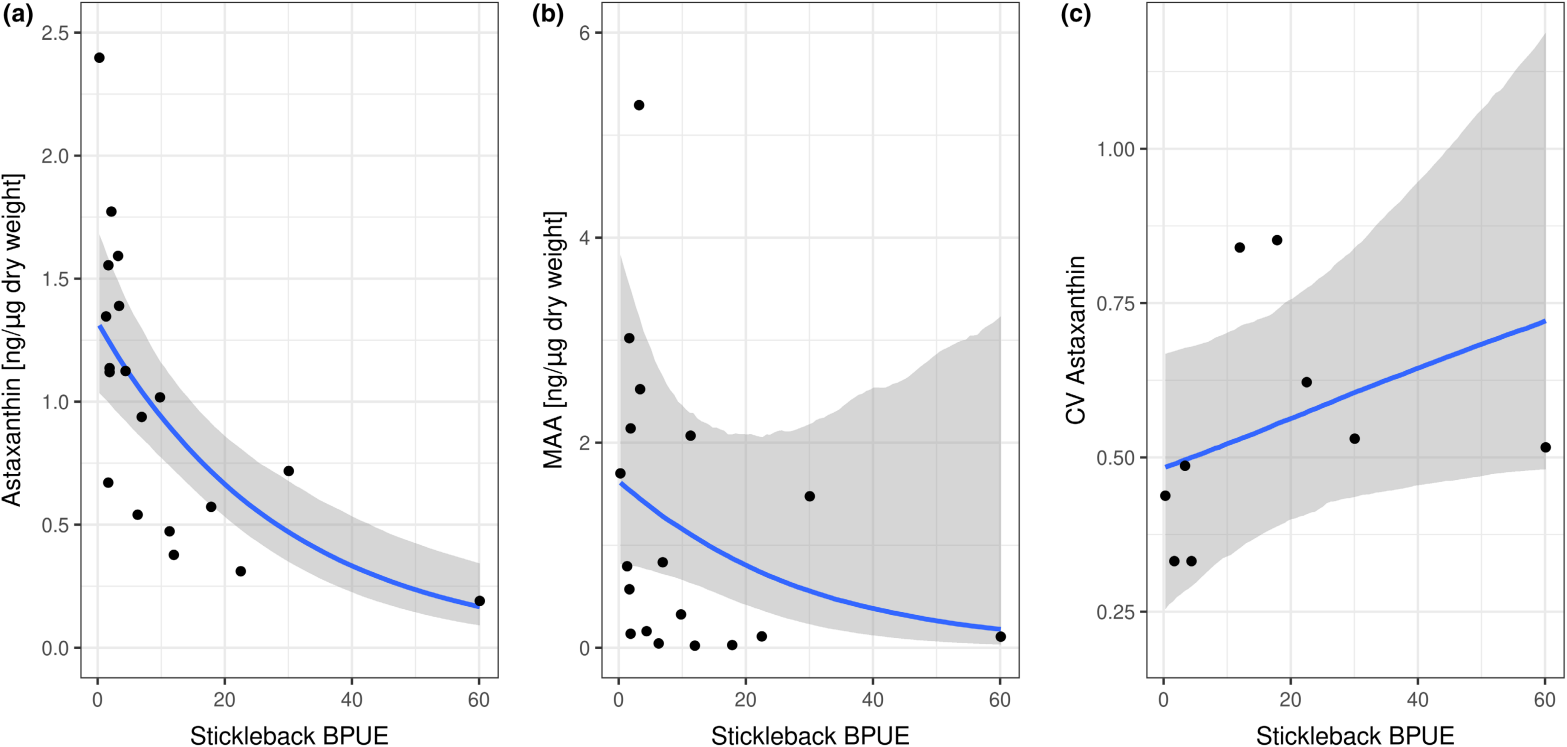
Effects of stickleback biomass per unit effort (BPUE) on (a) astaxanthin, (b) MAA, and (c) CV of astaxanthin. Dots represent the observed data points, and the shaded area shows 95% credible intervals

We found that the BPUE of stickleback was negatively associated with astaxanthin (estimate: −0.03, CI [−0.05; −0.02]) but not so with either MAA (estimate: −0.03, CI [−0.07; 0.02]) or the CV of astaxanthin (estimate: 0.00, CI [0.00; 0.01]; Figure 5; Table S5).

**FIGURE 6:**
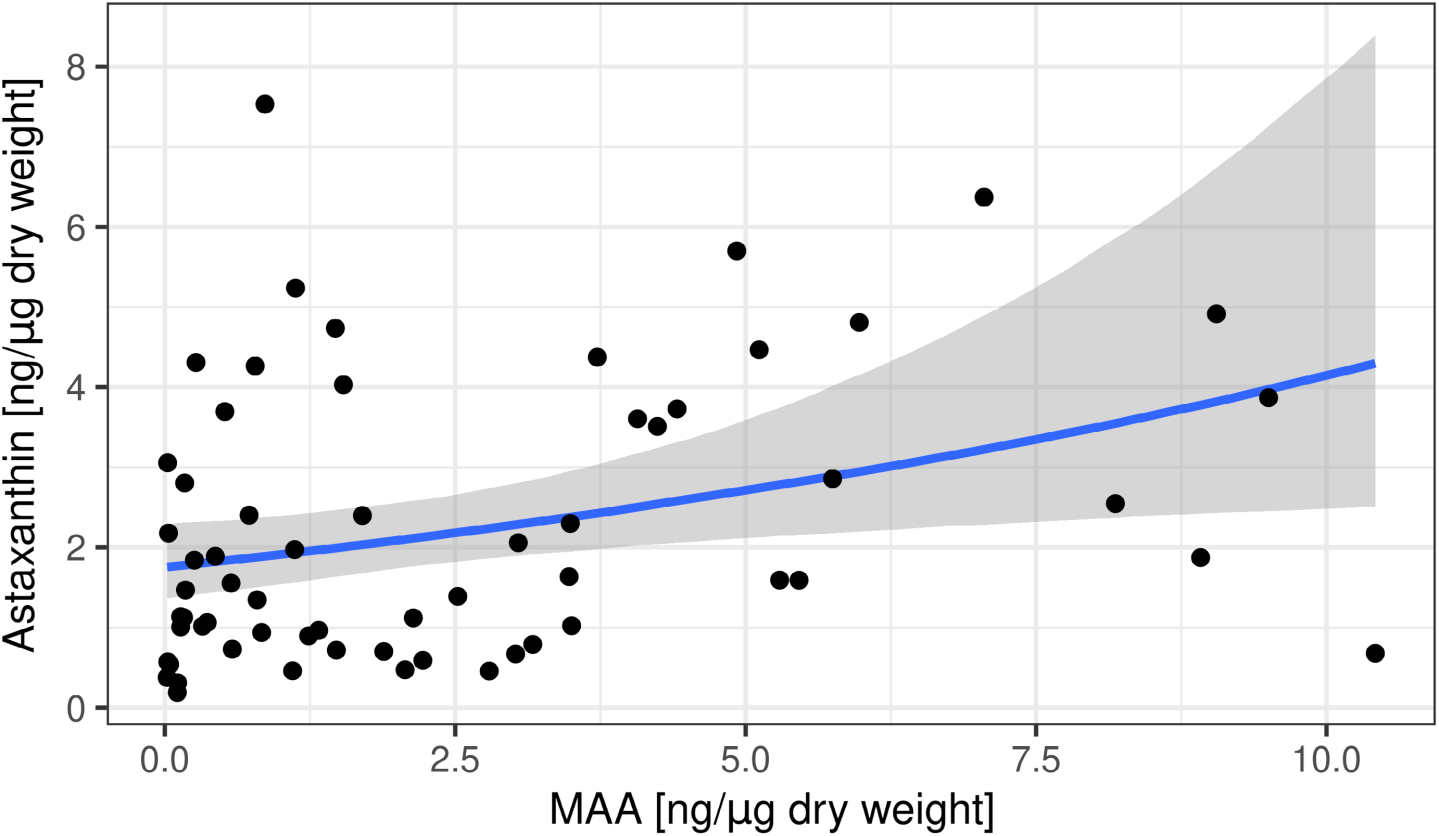
Relationship between astaxanthin and MAA contents. Dots represent the observed data points, and the shaded area shows 95% credible intervals

Lastly, we found a weak positive relationship between the two PC contents over all lakes (estimate: 0.09, CI [0.01–0.17]; Figure 6).

## 4 DISCUSSION

Prey species are often faced with multiple stressors in their environment that can affect their trait expression (Schmitz & Trussell, 2016). However, the trade‐offs associated with trait expression in natural populations are often poorly understood (Schmitz et al., 2017). In natural populations of copepods, multiple photoprotective compounds (PCs) that reduce the negative impacts of UVR damage exist, but our study is the first to demonstrate how the contents of these PCs depend on fish community composition, and the first to quantify individual‐level variation in astaxanthin for multiple natural populations of copepods.

Our comparative study of 73 Greenlandic lakes revealed that the predator community composition had large effects on the PCs of copepods (Figures 3 and 4). We found that the predator environment had greater explanatory power for copepod PCs than did the abiotic environment, which we characterized by light conditions (i.e., *K*d_UV_), DOC and chlorophyll *a*. While the path models showed an indirect effect of lake depth on astaxanthin and MAAs, this had a relatively minor effect size compared to the effects sizes due to predator communities (Table S3). Together, these results suggest that compared to the predator pressure, other environmental factors are of minor importance in explaining variation in the PCs of the copepods in our collection of lakes. In lakes with little or no predation risk, the light regime and the bottom‐up controls (such as algal food) are likely more important drivers of copepod PCs, as has been reported in previous work with a wider range of abiotic environmental conditions (Sommaruga, 2001; Tartarotti et al., 2017).

In lakes with sticklebacks, the astaxanthin contents in copepods were lower compared to values from fishless lakes (Figure 4a). Several studies have shown that the presence of fish decreased the expression of astaxanthin in copepods (Byron, 1982; Hansson, 2000; Hylander, Larsson, et al., 2009). Building on this work, our data show how the identity of fish species in a predator community can affect astaxanthin accumulation. Both stickleback and charr regularly feed on pelagic zooplankton and littoral macroinvertebrates. Stickleback, however, are more efficient zooplanktivores than charr, likely due to more specialized foraging traits that allow them to capture copepods (Jørgensen & Klemetsen, 1995; Schmid et al., 2019). Consistent with this, we found that across all lakes with stickleback, mean astaxanthin content was negatively correlated with stickleback biomass (Figure 5a), suggesting that variation in the biomass of efficient planktivorous species might help explain among‐lake variation in copepod coloration in other lakes and for other copepod species.

Fish community not only affected mean astaxanthin content among copepod populations but also the amount of individual variation within populations: the relative differences among individuals within a population were greater in lakes with stickleback (Figure 4c). Intrapopulation variation in astaxanthin expression is likely affected by factors such as heterogeneities in the light and resource environment (Cieri & Stearns, 1999), as well as predation risk by planktivorous fish. Our results are consistent with stickleback, and the predation risk they present, acting as agents of plasticity (Gvoždík & Boukal, 2021), and substantially lowering individual astaxanthin expression. It is unclear why stickleback might cause bigger differences among individuals at low content of astaxanthin expression (i.e., when predation risk is presumably the highest). One possibility is that non‐linear responses relating astaxanthin content to predation risk (or to UVR risk) generate a wider variation at high predation risk (Ramamonjisoa et al., 2019), but to test this we would need to rear copepods from multiple populations in common garden environments and measure astaxanthin reaction norms in response to variation in fish cues and UVR stress.

Even though MAAs are assumed to have no impact on predation risk (Hylander, Boeing, et al., 2009), we observed that MAA contents were slightly lower in lakes with only stickleback (Figure 4b), although this did not depend on stickleback biomass (Figure 5b). While there are few records of effective vision of fish in the absorbance band of MAAs (310–360 nm) (Leech & Johnsen, 2003), both stickleback and charr have been shown to use UVR for foraging (Parkyn & Hawryshyn, 2000; Rick et al., 2012). It has been suggested that copepods with high MAA contents may appear particularly dark against a UV‐rich background (Leech & Johnsen, 2003). Consistent with our results, one study confirmed that MAA contents were lower in a lake containing visually foraging fish compared to a fishless lake (Garcia et al., 2014). More research on the visual sensitivity of MAAs of different fish species would help clarify these possibilities.

Additionally, we found a slightly positive correlation between contents of astaxanthin and MAAs (Figure 6). If MAAs also increase the vulnerability of copepods to fish predation, this might help explain the observed positive correlation between astaxanthin and MAAs. In these oligotrophic lakes, it might simply be necessary to allocate as many resources as possible and rely on multiple pathways to prevent photodamage (Tartarotti et al., 2004). Another explanation for this positive relationship could involve varying resource abundances enabling some populations to invest in both pathways without limiting resource allocation to either trait (Stearns, 1989). Although we observed a limited range of variation in total algal biomass (Table 1), we did not characterize variation in algal composition which might underlie variation in the availability of MAAs and astaxanthin (Hylander, Boeing, et al., 2009; Sommaruga, 2010; Stuart‐Fox et al., 2021). Finally, this positive correlation could be driven by different behavioral adaptations of populations to UVR and predators. As we took zooplankton samples over the entire water column, populations may vary in their water depth utilization and their degree of refuge use (e.g., vertical migration) (Hylander, Larsson, et al., 2009).

In conclusion, we show that variation in the expression of PCs of *L*. *minutus* can be explained mainly by the species identity of planktivorous fish. Astaxanthin and MAAs showed lower contents when sticklebacks were present, whereas the CV of astaxanthin showed highest values in these lakes. This implies that the regulation of different pathways of photoprotection in copepods can depend on the presence and density of specific predator species. In addition, contents of astaxanthin and MAAs were positively correlated across the surveyed populations. These patterns provide new insights into how predator community composition can affect the PCs of copepods in natural settings. More generally, our results highlight that the community context of trait expression clearly matters for a trait that is mediated by multiple biotic and abiotic factors.

## CONFLICT OF INTEREST

All authors declare no conflict of interest.

## AUTHOR CONTRIBUTIONS

**Rebecca Oester:**Conceptualization (equal); Data curation (lead); Formal analysis (lead); Investigation (lead); Methodology (lead); Project administration (equal); Resources (supporting); Validation (equal); Visualization (lead); Writing – original draft (lead); Writing – review & editing (equal). **Ryan Greenway:** Conceptualization (supporting); Investigation (supporting); Supervision (supporting); Writing – original draft (supporting); Writing – review & editing (equal). **Marvin Moosmann:** Conceptualization (supporting); Investigation (supporting); Supervision (supporting); Writing – original draft (supporting); Writing – review & editing (equal). **Riben Sommaruga:** Conceptualization (supporting); Methodology (supporting); Resources (supporting); Validation (supporting); Writing – review & editing (equal). **Barbara Tartarotti:** Conceptualization (supporting); Formal analysis (supporting); Investigation (supporting); Methodology (supporting); Resources (supporting); Supervision (supporting); Validation (supporting); Writing – review & editing (equal). **Jakob Brodersen:** Conceptualization (supporting); Investigation (supporting); Methodology (supporting); Resources (supporting); Validation (supporting); Writing – review & editing (equal). **Blake Matthews:** Conceptualization (equal); Data curation (supporting); Formal analysis (supporting); Funding acquisition (lead); Investigation (supporting); Methodology (supporting); Project administration (equal); Resources (lead); Supervision (lead); Validation (supporting); Writing – original draft (supporting); Writing – review & editing (equal).

### OPEN RESEARCH BADGES

This article has earned an Open Data Badge for making publicly available the digitally‐shareable data necessary to reproduce the reported results. The data is available at https://doi.org/10.5061/dryad.hqbzkh1gr.

## Supporting information

Supplementary Material

## Data Availability

The data that support the findings of this study are openly available on Dryad: https://doi.org/10.5061/dryad.hqbzkh1gr.
