# Supplementary material for: The influence of predator community composition on photoprotective traits of copepods
Source: Ecol Evol. 2022 Apr 24;12(4):e8862. doi: 10.1002/ece3.8862 (PMC9035585; doi:10.1002/ece3.8862)
Supplement: Supplementary file 1 — Supplementary Material [file ECE3-12-e8862-s001.docx]

**Supporting Information**

Table S1: Main characteristics of the studied lakes grouped by fish community; mean (range). *K*d values represent the extinction coefficient of the incoming photosynthetic active radiation (PAR) or ultraviolet radiation (UVR). Based on the *K*ds, the depths where only 1 % radiation penetrated the water column (z_1%_) were calculated (z_1%UVR_ =4.605/*K*d_UVR_). The ratio of z_1%UVR_ and the maximum depth of the lake represent the refuge from UVR for copepods, with high values reflecting less refuge.

| Fish community | Area  [ha] | Elevation  [m a.s.l.] | Depth  [m] | Chl *a*  [μg/l] | *K*d_PAR_  [m^-1^] | *K*d_UVR_  [m^-1^] | DOC  [mg/l] |
| --- | --- | --- | --- | --- | --- | --- | --- |
| No Fish (28) | 1.5  (0.2-13.7) | 164.9  (3.4-304.5) | 2.8  (0.8-7.0) | 1.1  (0.1-4.3) | 0.6  (0.2-2.4) | 2.7  (0.3-9.8) | 4.6  (1.9-6.7) |
| Charr (8) | 7.7  (1.4-18.0) | 157.8  (110.0-290.0) | 8.7  (1.0-19.0 | 1.2  (0.3-2.8) | 0.8  (0.1-2.4) | 2.0  (0.4-5.2) | 3.4  (2.2-4.5) |
| Stickleback +  Charr (21) | 15.2  (1.5-51.8) | 52.8  (4.0-139.0) | 11.0  (1.0-36.0) | 1.3  (0.2-4.1) | 0.5  (0.2-2.6) | 1.2  (0.2-1.8) | 3.8  (2.2-4.5) |
| Stickleback (16) | 7.1  (0.3-64.5) | 59.6  (13.7-170.7) | 8.2  (1.0-49.5) | 1.8  (0.2-9.3) | 0.5  (0.1-2.0) | 2.4  (0.3-8.5) | 4.1  (2.2-6.8) |

Table S2: Main characteristics of the zooplankton communities of the studied lakes grouped by fish community; mean (range). Sizes for *L. minutus* represent prosomal lengths.

| Fish community | Total Biomass [µg/l] | % Biomass  *L. minutus* | Abundance *L. minutus*  [#/l] | Community size [mm] | Size *L. minutus* [mm] | # Taxa |
| --- | --- | --- | --- | --- | --- | --- |
| No Fish | 389.39  (18.16-2480.04) | 67.41  (0.65-99.73) | 71.37  (4.12-216.43) | 0.89  (0.48-2.6) | 0.67  (0.54-0.82) | 7.95  (2-14) |
| Charr | 703.23  (69.48-2980.03) | 30.46  (7.91-74.13) | 45.25  (14.34-83.12) | 0.74  (0.49-1.71) | 0.63  (0.51-0.75) | 5.88  (2-9) |
| Stickleback+  Charr | 103.87  (0.09-560.79) | 60.99  (10.25-99.64) | 15.54  (0.02-55.41) | 0.50  (0.30-0.77) | 0.65  (0.44-0.81) | 5.33  (3-16) |
| Stickleback | 31.58  (0.53-96.79) | 79.50  (23.30-100.00) | 7.98  (0.16-24.77) | 0.43  (0.26-0.82) | 0.66  (0.45-0.82) | 4.06  (1-9) |

Table S3: Summary output of the path models.

| Variable | Estimate | Est.Error | CI l-95% | CI u-95 | Rhat | BulkESS | TailEss |
| --- | --- | --- | --- | --- | --- | --- | --- |
| Astaxanthin |  |  |  |  |  |  |  |
| Astaxanthin Intercept | 0.29 | 0.08 | 0.14 | 0.45 | 1.00 | 14357 | 13991 |
| Chlorophyll Intercept | -0.22 | 0.14 | -0.49 | 0.05 | 1.00 | 16006 | 13740 |
| DOC Intercept | 0.10 | 0.13 | -0.16 | 0.35 | 1.00 | 16288 | 14036 |
| KdUV Intercept | 0.12 | 0.13 | -0.15 | 0.38 | 1.00 | 16854 | 14657 |
| C Fish Intercept | 0.01 | 0.67 | -1.26 | 1.38 | 1.00 | 8764 | 10108 |
| SBC Fish Intercept | 0.32 | 0.65 | -0.89 | 1.63 | 1.00 | 8954 | 9911 |
| SB Fish Intercept | 0.44 | 0.63 | -0.71 | 1.76 | 1.00 | 7772 | 9191 |
| Astaxanthin<- FishC | -0.12 | 0.15 | -0.41 | 0.17 | 1.00 | 16987 | 14489 |
| Astaxanthin<- FishSBC | -0.64 | 0.15 | -0.94 | -0.35 | 1.00 | 12620 | 14429 |
| Astaxanthin<- FishSB | -0.71 | 0.13 | -0.96 | -0.45 | 1.00 | 16231 | 15541 |
| Astaxanthin<- MaxDepth | -0.03 | 0.12 | -0.27 | 0.22 | 1.00 | 16000 | 14009 |
| Astaxanthin<- KdUV | -0.10 | 0.10 | -0.29 | 0.08 | 1.00 | 19866 | 12499 |
| Astaxanthin<- DOC | 0.07 | 0.09 | -0.11 | 0.26 | 1.00 | 20732 | 14235 |
| Astaxanthin<- Chlorophyll | -0.07 | 0.09 | -0.25 | 0.12 | 1.00 | 16673 | 14263 |
| Chlorophyll<-FishC | 0.16 | 0.26 | -0.36 | 0.67 | 1.00 | 18866 | 15940 |
| Chlorophyll<-FishSBC | 0.54 | 0.25 | 0.06 | 1.04 | 1.00 | 15024 | 14733 |
| Chlorophyll<-FishSB | 0.52 | 0.22 | 0.07 | 0.95 | 1.00 | 18811 | 14610 |
| Chlorophyll<-MaxDepth | -0.43 | 0.21 | -0.85 | -0.01 | 1.00 | 17472 | 15134 |
| DOC<-FishC | -0.32 | 0.25 | -0.80 | 0.16 | 1.00 | 18321 | 14336 |
| DOC<-FishSBC | 0.03 | 0.23 | -0.42 | 0.50 | 1.00 | 15889 | 14854 |
| DOC<-FishSB | -0.05 | 0.21 | -0.46 | 0.36 | 1.00 | 19646 | 15015 |
| DOC<-MaxDepth | -0.36 | 0.20 | -0.76 | 0.05 | 1.00 | 18514 | 15090 |
| KdUV<-FishC | -0.10 | 0.26 | -0.61 | 0.40 | 1.00 | 18953 | 14337 |
| KdUV<-FishSBC | -0.25 | 0.24 | -0.74 | 0.22 | 1.00 | 15194 | 14934 |
| KdUV<-FishSB | 0.04 | 0.22 | -0.39 | 0.48 | 1.00 | 19980 | 15061 |
| KdUV<-MaxDepth | -0.20 | 0.21 | -0.61 | 0.20 | 1.00 | 16404 | 14386 |
| C Fish<-MaxDepth | 8.72 | 2.74 | 3.81 | 14.54 | 1.00 | 7060 | 8251 |
| SBC Fish<-MaxDepth | 10.14 | 2.73 | 5.33 | 16.01 | 1.00 | 6904 | 8058 |
| SB Fish<-MaxDepth | 6.73 | 2.66 | 1.90 | 12.38 | 1.00 | 6808 | 8023 |
| MAA |  |  |  |  |  |  |  |
| MAA Intercept | 0.14 | 0.12 | -0.09 | 0.37 | 1.00 | 12807 | 14080 |
| Chlorophyll Intercept | -0.20 | 0.15 | -0.49 | 0.09 | 1.00 | 13917 | 13035 |
| DOC Intercept | 0.05 | 0.14 | -0.22 | 0.33 | 1.00 | 13498 | 13096 |
| KdUV Intercept | 0.10 | 0.15 | -0.19 | 0.39 | 1.00 | 13413 | 13804 |
| C Fish Intercept | -0.10 | 0.71 | -1.46 | 1.37 | 1.00 | 7823 | 8757 |
| SBC Fish Intercept | 0.43 | 0.67 | -0.81 | 1.80 | 1.00 | 7352 | 8743 |
| SB Fish Intercept | 0.45 | 0.66 | -0.78 | 1.81 | 1.00 | 6577 | 7502 |
| MAA<- FishC | -0.42 | 0.22 | -0.85 | 0.02 | 1.00 | 16338 | 14750 |
| MAA<- FishSBC | -0.36 | 0.21 | -0.79 | 0.06 | 1.00 | 11673 | 14240 |
| MAA<- FishSB | -0.48 | 0.20 | -0.87 | -0.09 | 1.00 | 13959 | 14163 |
| MAA<- MaxDepth | 0.15 | 0.18 | -0.21 | 0.52 | 1.00 | 14549 | 14565 |
| MAA<- KdUV | 0.04 | 0.14 | -0.24 | 0.32 | 1.00 | 16943 | 14377 |
| MAA<- DOC | 0.00 | 0.14 | -0.27 | 0.26 | 1.00 | 17918 | 14438 |
| MAA<- Chlorophyll | -0.06 | 0.14 | -0.33 | 0.21 | 1.00 | 14517 | 14477 |
| Chlorophyll<-FishC | 0.11 | 0.28 | -0.45 | 0.66 | 1.00 | 15621 | 14909 |
| Chlorophyll<-FishSBC | 0.52 | 0.26 | 0.01 | 1.03 | 1.00 | 13005 | 13609 |
| Chlorophyll<-FishSB | 0.56 | 0.24 | 0.10 | 1.03 | 1.00 | 16200 | 14688 |
| Chlorophyll<-MaxDepth | -0.45 | 0.22 | -0.89 | 0.00 | 1.00 | 15484 | 14567 |
| DOC<-FishC | -0.24 | 0.27 | -0.77 | 0.29 | 1.00 | 15676 | 14214 |
| DOC<-FishSBC | 0.07 | 0.24 | -0.41 | 0.55 | 1.00 | 12948 | 14362 |
| DOC<-FishSB | -0.07 | 0.23 | -0.51 | 0.38 | 1.00 | 16840 | 14942 |
| DOC<-MaxDepth | -0.36 | 0.21 | -0.77 | 0.06 | 1.00 | 14953 | 14707 |
| KdUV<-FishC | -0.01 | 0.29 | -0.57 | 0.55 | 1.00 | 14476 | 13841 |
| KdUV<-FishSBC | -0.23 | 0.26 | -0.74 | 0.27 | 1.00 | 12217 | 13758 |
| KdUV<-FishSB | 0.08 | 0.24 | -0.38 | 0.54 | 1.00 | 15747 | 13198 |
| KdUV<-MaxDepth | -0.24 | 0.22 | -0.68 | 0.20 | 1.00 | 14057 | 13859 |
| C Fish<-MaxDepth | 8.87 | 2.84 | 3.77 | 14.86 | 1.00 | 5859 | 6644 |
| SBC Fish<-MaxDepth | 10.07 | 2.78 | 5.12 | 15.96 | 1.00 | 5551 | 6372 |
| SB Fish<-MaxDepth | 6.90 | 2.73 | 1.93 | 12.60 | 1.00 | 5645 | 6790 |
| CV Astaxanthin |  |  |  |  |  |  |  |
| CV Astaxanthin Intercept | -0.30 | 0.24 | -0.78 | 0.16 | 1.00 | 12497 | 12855 |
| Chlorophyll Intercept | -0.26 | 0.23 | -0.71 | 0.18 | 1.00 | 11350 | 12707 |
| DOC Intercept | -0.13 | 0.24 | -0.60 | 0.34 | 1.00 | 12144 | 11705 |
| KdUV Intercept | -0.12 | 0.20 | -0.52 | 0.28 | 1.00 | 10804 | 11298 |
| C Fish Intercept | 0.25 | 0.81 | -1.27 | 1.92 | 1.00 | 9192 | 9438 |
| SBC Fish Intercept | 0.62 | 0.77 | -0.82 | 2.24 | 1.00 | 9033 | 8666 |
| SB Fish Intercept | 0.31 | 0.80 | -1.20 | 1.94 | 1.00 | 8441 | 8644 |
| CV Astaxanthin<- FishC | 0.08 | 0.35 | -0.62 | 0.76 | 1.00 | 13682 | 13675 |
| CV Astaxanthin<- FishSBC | 0.27 | 0.39 | -0.51 | 1.05 | 1.00 | 10377 | 12029 |
| CV Astaxanthin<- FishSB | 0.80 | 0.37 | 0.06 | 1.53 | 1.00 | 15377 | 13460 |
| CV Astaxanthin<- MaxDepth | 0.28 | 0.30 | -0.29 | 0.88 | 1.00 | 12921 | 12441 |
| CV Astaxanthin<- KdUV | -0.23 | 0.50 | -1.21 | 0.75 | 1.00 | 11148 | 11112 |
| CV Astaxanthin<- DOC | 0.05 | 0.24 | -0.42 | 0.53 | 1.00 | 18241 | 13779 |
| CV Astaxanthin<- Chlorophyll | 0.16 | 0.42 | -0.67 | 1.00 | 1.00 | 11223 | 11630 |
| Chlorophyll<-FishC | 0.14 | 0.35 | -0.54 | 0.84 | 1.00 | 12292 | 13094 |
| Chlorophyll<-FishSBC | 0.39 | 0.33 | -0.25 | 1.03 | 1.00 | 11743 | 13504 |
| Chlorophyll<-FishSB | 0.52 | 0.32 | -0.10 | 1.16 | 1.00 | 13459 | 13031 |
| Chlorophyll<-MaxDepth | -0.44 | 0.26 | -0.96 | 0.08 | 1.00 | 15287 | 14042 |
| DOC<-FishC | 0.02 | 0.36 | -0.71 | 0.73 | 1.00 | 12921 | 12791 |
| DOC<-FishSBC | 0.39 | 0.34 | -0.29 | 1.07 | 1.00 | 12426 | 11916 |
| DOC<-FishSB | 0.08 | 0.34 | -0.58 | 0.75 | 1.00 | 14618 | 12765 |
| DOC<-MaxDepth | -0.44 | 0.27 | -0.98 | 0.10 | 1.00 | 16318 | 14479 |
| KdUV<-FishC | 0.03 | 0.30 | -0.57 | 0.63 | 1.00 | 12433 | 12315 |
| KdUV<-FishSBC | -0.08 | 0.28 | -0.65 | 0.49 | 1.00 | 11827 | 12269 |
| KdUV<-FishSB | 0.66 | 0.28 | 0.10 | 1.21 | 1.00 | 14424 | 12831 |
| KdUV<-MaxDepth | -0.25 | 0.23 | -0.70 | 0.20 | 1.00 | 15481 | 13616 |
| C Fish<-MaxDepth | 4.89 | 2.22 | 1.04 | 9.75 | 1.00 | 8102 | 9077 |
| SBC Fish<-MaxDepth | 4.93 | 2.15 | 1.20 | 9.63 | 1.00 | 7429 | 9334 |
| SB Fish<-MaxDepth | 2.45 | 2.21 | -1.66 | 7.05 | 1.00 | 7805 | 7967 |

Table S4: Summary output of the models assessing the influence of fish community and lake depth on astaxanthin, MAAs and the CV of astaxanthin.

| Variable | Estimate | Est.Error | CI l-95% | CI u-95% | Rhat | Bulk_ESS | Tail_ESS |
| --- | --- | --- | --- | --- | --- | --- | --- |
| Astaxanthin |  |  |  |  |  |  |  |
| Intercept | 1.69 | 0.18 | 1.34 | 2.07 | 1.00 | 1081 | 1348 |
| FishC | -0.57 | 0.33 | -1.21 | 0.09 | 1.00 | 1639 | 1905 |
| FishSBC | -1.74 | 0.25 | -2.24 | -1.26 | 1.00 | 1467 | 2094 |
| FishSB | -1.77 | 0.28 | -2.31 | -1.22 | 1.00 | 1407 | 1866 |
| MaxDepth | -0.14 | 0.06 | -0.27 | -0.02 | 1.00 | 964 | 1333 |
| FishC:MaxDepth | 0.14 | 0.07 | 0.00 | 0.27 | 1.00 | 962 | 1258 |
| FishSBC:MaxDepth | 0.15 | 0.06 | 0.03 | 0.28 | 1.00 | 956 | 1250 |
| FishSB:MaxDepth | 0.12 | 0.07 | -0.02 | 0.26 | 1.00 | 996 | 1429 |
| MAA |  |  |  |  |  |  |  |
| Intercept | 1.33 | 0.50 | 0.34 | 2.33 | 1.00 | 939 | 1617 |
| FishC | -1.18 | 1.44 | -3.78 | 1.89 | 1.00 | 1719 | 1901 |
| FishSBC | -0.47 | 0.63 | -1.71 | 0.78 | 1.00 | 1079 | 1754 |
| FishSB | -1.42 | 0.70 | -2.77 | 0.03 | 1.00 | 1194 | 1772 |
| MaxDepth | 0.03 | 0.17 | -0.29 | 0.41 | 1.00 | 848 | 1534 |
| FishC:MaxDepth | 0.00 | 0.21 | -0.44 | 0.39 | 1.00 | 873 | 1202 |
| FishSBC:MaxDepth | -0.03 | 0.18 | -0.41 | 0.29 | 1.00 | 844 | 1352 |
| FishSB:MaxDepth | -0.02 | 0.19 | -0.42 | 0.32 | 1.00 | 854 | 1335 |
| CV of Astaxanthin |  |  |  |  |  |  |  |
| Intercept | 0.29 | 0.06 | 0.15 | 0.40 | 1.00 | 3796 | 3067 |
| FishC | 0.12 | 0.08 | 0.01 | 0.30 | 1.00 | 2695 | 1666 |
| FishSBC | 0.15 | 0.09 | 0.02 | 0.33 | 1.00 | 2261 | 1540 |
| FishSB | 0.18 | 0.11 | 0.02 | 0.42 | 1.00 | 2314 | 1680 |
| MaxDepth | 0.00 | 0.00 | 0.00 | 0.01 | 1.00 | 2694 | 1572 |
| FishC:MaxDepth | 0.01 | 0.00 | 0.00 | 0.02 | 1.00 | 2689 | 1821 |
| FishSBC:MaxDepth | 0.01 | 0.00 | 0.00 | 0.02 | 1.00 | 3063 | 2074 |
| FishSB:MaxDepth | 0.02 | 0.01 | 0.00 | 0.05 | 1.00 | 1713 | 828 |

Table S5: Summary output of the models assessing the influence of stickleback biomass per unit effort on astaxanthin, MAAs and the CV of astaxanthin.

| Variable | Estimate | Est.Error | CI l-95% | CI u-95% | Rhat | Bulk_ESS | Tail_ESS |
| --- | --- | --- | --- | --- | --- | --- | --- |
| Astaxanthin |  |  |  |  |  |  |  |
| Intercept | 0.28 | 0.13 | 0.04 | 0.53 | 1.00 | 3037 | 2296 |
| SBBPUE | -0.03 | 0.01 | -0.05 | -0.02 | 1.00 | 3331 | 2628 |
| MAA |  |  |  |  |  |  |  |
| Intercept | 0.50 | 0.38 | -0.20 | 1.30 | 1.00 | 3161 | 2260 |
| SBBPUE | -0.03 | 0.02 | -0.07 | 0.02 | 1.00 | 2657 | 2082 |
| CV of Astaxanthin | |  |  |  |  |  |  |
| Intercept | 0.47 | 0.10 | 0.25 | 0.66 | 1.00 | 1925 | 1649 |
| SBBPUE | 0.00 | 0.00 | 0.00 | 0.01 | 1.00 | 1627 | 1085 |
